# Crosstalk Between ATP-P_2X7_ and Adenosine A_2A_ Receptors Controlling Neuroinflammation in Rats Subject to Repeated Restraint Stress

**DOI:** 10.3389/fncel.2021.639322

**Published:** 2021-03-01

**Authors:** Liliana Dias, Cátia R. Lopes, Francisco Q. Gonçalves, Ana Nunes, Daniela Pochmann, Nuno J. Machado, Angelo R. Tomé, Paula Agostinho, Rodrigo A. Cunha

**Affiliations:** ^1^CNC—Center for Neuroscience and Cell Biology, University of Coimbra, Coimbra, Portugal; ^2^Department of Life Sciences, Faculty of Sciences and Technology, University of Coimbra, Coimbra, Portugal; ^3^Faculty of Medicine, University of Coimbra, Coimbra, Portugal

**Keywords:** ATP P_2X7_ receptor, adenosine A_2A_ receptor, stress, behavior, microglia, neuroinflammation, synaptic plasticity

## Abstract

Depressive conditions precipitated by repeated stress are a major socio-economical burden in Western countries. Previous studies showed that ATP-P_2X7_ receptors (P_2X7_R) and adenosine A_2A_ receptors (A_2A_R) antagonists attenuate behavioral modifications upon exposure to repeated stress. Since it is unknown if these two purinergic modulation systems work independently, we now investigated a putative interplay between P_2X7_R and A_2A_R. Adult rats exposed to restraint stress for 14 days displayed an anxious (thigmotaxis, elevated plus maze), depressive (anhedonia, increased immobility), and amnesic (modified Y maze, object displacement) profile, together with increased expression of Iba-1 (a marker of microglia “activation”) and interleukin-1β (IL1β) and tumor necrosis factor α (TNFα; proinflammatory cytokines) and an up-regulation of P_2X7_R (mRNA) and A_2A_R (receptor binding) in the hippocampus and prefrontal cortex. All these features were attenuated by the P_2X7_R-preferring antagonist brilliant blue G (BBG, 45 mg/kg, i.p.) or by caffeine (0.3 g/L, *p.o*.), which affords neuroprotection through A_2A_R blockade. Notably, BBG attenuated A_2A_R upregulation and caffeine attenuated P_2X7_R upregulation. In microglial N9 cells, the P_2X7_R agonist BzATP (100 μM) or the A_2A_R agonist CGS26180 (100 nM) increased calcium levels, which was abrogated by the P_2X7_R antagonist JNJ47965567 (1 μM) and by the A_2A_R antagonist SCH58261 (50 nM), respectively; notably JNJ47965567 prevented the effect of CGS21680 and the effect of BzATP was attenuated by SCH58261 and increased by CGS21680. These results provide the first demonstration of a functional interaction between P_2X7_R and A_2A_R controlling microglia reactivity likely involved in behavioral adaptive responses to stress and are illustrative of a cooperation between the two arms of the purinergic system in the control of brain function.

## Introduction

Depression represents the major burden of disease in Europe (Andlin-Sobocki et al., 2005) and the constellation of mood alterations associated with depression can be recapitulated in animal models repeatedly exposed to different stressors (de Kloet et al., 2005; Berton et al., 2012). The use of animal models converges with imaging studies to identify modifications of different brain regions, such as the hippocampus, prefrontal, and limbic cortices, that are associated with mood dysfunction (de Kloet et al., 2005) and provide compelling evidence for the involvement of neuroinflammation (Rial et al., 2016; Deng et al., 2020; Troubat et al., 2021) and of synaptic dysfunction (Duman and Aghajanian, 2012; Vose and Stanton, 2017) as key processes in the etiology of major depression. However, the identification of molecular systems that may be targeted to correct depressive symptoms has still failed to yield novel and effective anti-depressants (Ménard et al., 2016).

One candidate system is operated by purines, which fulfill numerous roles controlling neuronal communication, neuron-glia communication, and neuroinflammation (Agostinho et al., 2020). ATP is a danger signal in the brain (Rodrigues et al., 2015) and one of its receptors, P_2X7_ receptors (P_2X7_R), has been associated with mood dysfunction (reviewed in Ribeiro et al., 2019; Illes et al., 2020), based on the association of particular P_2X7_R haplotypes with depression (Czamara et al., 2018) and with the ability of genetic deletion or pharmacological antagonism of P_2X7_R to control mood dysfunction in different animal models of repeated stress (Iwata et al., 2016; Yue et al., 2017; Farooq et al., 2018; Aricioglu et al., 2019). The mechanism underlying the impact of P_2X7_R on mood is still undefined, but the control of glia, mainly microglia, which contributes to the build-up of neuroinflammation, stems as a promising candidate mechanism (Yue et al., 2017; Bhattacharya and Jones, 2018). Together with possible neuronal effects of P_2X7_R, the control of neuroinflammation can account for the general neuroprotective properties of P_2X7_R antagonists, such as the blood-brain barrier-permeant drug, brilliant blue G (BBG; Díaz-Hernández et al., 2009, 2012; Arbeloa et al., 2012; Carmo et al., 2014; Wang et al., 2015; Yue et al., 2017; Farooq et al., 2018; Aricioglu et al., 2019).

The purinergic system is particularly enticing since it encompasses two parallel signaling systems: one involving ATP and P_2_R and the other involving the dephosphorylation product of ATP, adenosine, which acts on P_1_ or adenosine receptors, mainly inhibitory A_1_ receptors and facilitatory A_2A_ receptors (A_2A_R) in the brain (Fredholm et al., 2005). The extracellular conversion of ATP into adenosine is mediated by ectonucleotidases (Cunha, 2001; Zimmermann et al., 2012) and we have shown that the extracellular formation of ATP-derived adenosine is selectively associated with the activation of neuronal A_2A_R (Rebola et al., 2008; Augusto et al., 2013; Carmo et al., 2019; Gonçalves et al., 2019), as well as with A_2A_R located in other cell types (e.g., Deaglio et al., 2007; Flögel et al., 2012; Flores-Santibáñez et al., 2015; Mahmut et al., 2015; Meng et al., 2019). A_2A_R are mainly located in synapses (Rebola et al., 2005), but also control microglia and neuroinflammation (Orr et al., 2009; Rebola et al., 2011; Madeira et al., 2016; Duarte et al., 2019) to robustly impact neurodegeneration (reviewed in Cunha, 2016). Both selective A_2A_R antagonists and the non-selective adenosine receptor antagonist caffeine (Fredholm et al., 1999), can control mood and memory alterations in rodents exposed to repeated stress (Yamada et al., 2013; Kaster et al., 2015), as per the mood normalizing properties afforded by the intake of caffeine in humans (reviewed in Grosso et al., 2016) and the association of A_2A_R polymorphisms with anxiety and depression (Hamilton et al., 2004; Hohoff et al., 2010; Oliveira et al., 2019).

Thus, the available evidence indicates P_2X7_R as well as A_2A_R as major players in the control of mood dysfunction, with both receptors systems undergoing an up-regulation in animal models exposed to repeated stress (Cunha et al., 2006; Kongsui et al., 2014; Kaster et al., 2015; Aricioglu et al., 2019). However, it has never been explored if there is any interplay between both receptors systems in the control of mood dysfunction. As a first step to test the existence of such an interplay, we now exploited a rat model of repeated restraint stress to test if P_2X7_R blockade with BBG would impact A_2A_R up-regulation and, conversely, if caffeine blockade of A_2A_R could interfere with P_2X7_R up-regulation.

## Materials and Methods

### Animals

Male Wistar rats (adults, 220–250 g, *n* = 78: 18 controls treated with vehicle, nine controls treated with BBG, nine controls treated with caffeine; 18 stressed treated with vehicle, nine stressed treated with BBG, nine stressed treated with caffeine, six for electrophysiology) were obtained from Charles River (Barcelona, Spain) and were maintained at 23–25°C, with 12 h light / 12 h dark cycle and standard chow and tap water *ad libitum*. All procedures in this study were conducted following the principles and procedures outlined as “3Rs” in the guidelines of the European Union (2010/63/EU), FELASA, and ARRIVE, and were approved by the Portuguese Ethical Committee (DGAV) and by the Institution’s Ethics’ Committee (ORBEA 238-2019/14102019). Since the behavioral alterations caused by this protocol of restraint stress were so far only validated in male rats, the “3Rs” guidelines imposed the use of only male rats to obtain the first proof-of-concept supporting the existence of any interaction between P_2X7_R and A_2A_R.

### *In vivo* Drug Treatments

As done previously (Carmo et al., 2014), the blood-brain barrier-permeant and efficacious P_2X7_R antagonist brilliant blue G (BBG, 45 mg/kg dissolved in saline; from Sigma–Aldrich, Portugal) or saline were administered intraperitoneally every 48 h at 7 PM, starting 3 days before the protocol of restraint stress, until the sacrifice of the animals. The tested dose of BBG has previously been shown to yield a brain concentration of 200–220 nM (Díaz-Hernández et al., 2012), which is within the effective and selective range of BBG towards central P_2X7_R and is without evident side-effects in control rodents (Donnelly-Roberts and Jarvis, 2007).

Caffeine (Sigma, Portugal) was administered through the drinking water as previously reported (Duarte et al., 2009; Cognato et al., 2010) at a dose (0.3 g/L) estimated to correspond to a daily intake of 3–4 cups of coffee by humans (Fredholm et al., 1999), which rodents consume without modification of their water intake (Duarte et al., 2009, 2012; Silva et al., 2013). This yields a concentration of *circa* 30 μM in the brain parenchyma (Costenla et al., 2010; Silva et al., 2013), which selectively targets adenosine receptors (Lopes et al., 2019) and mimics the neuroprotective impact of A_2A_R antagonists, rather than of A_1_R (Cunha et al., 2006; Dall’Igna et al., 2007), namely in animal models of stress and depression (Kaster et al., 2015; Machado et al., 2017). Caffeine intake was allowed only overnight (7 PM-7 AM), starting 3 days before the protocol of restraint stress, until the sacrifice of the animals and this repeated exposure to caffeine is expected to afford neuroprotection without major modification of behavioral or physiological parameters in control rodents (Duarte et al., 2009; Yang et al., 2009; Cognato et al., 2010).

### Restraint Stress

The stress model used consisted of a repeated physical restraint of rats, as done previously (Cunha et al., 2006). The rats were individually placed in a room adjacent to their colony in an independent plastic compartment and immobilized in a 25 × 7 cm plastic bottle, with a plastic taper on the outside and a 1 cm hole at one end for breathing. After the termination of each daily restraint stress session, the rats were returned to their home cages. The schedule of sub-chronic restraint stress consisted of a daily 4 h immobilization period (between 10 AM and 4 PM) during 14 consecutive days, the time previously defined to be required to cause stable behavioral modifications for at least 1 week in adult male rats (Cunha et al., 2006). Control age-matched rats were handled as their tested littermates except that they were not isolated or immobilized.

### Behavioral Evaluation

**Figure 1:**
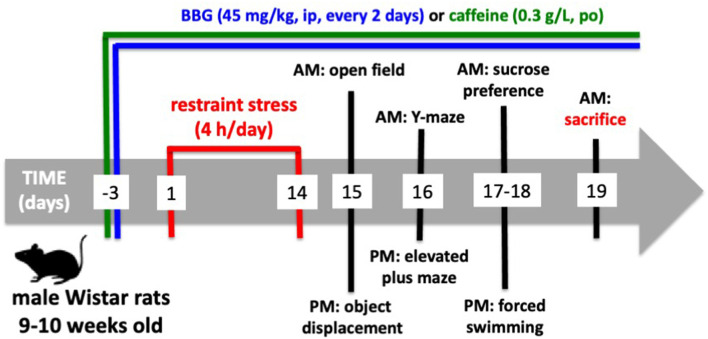
Timeline of the experiments.

Behavioral tests were carried out from 9 AM until 4 PM on the 15^th^ until the 18^th^ day after beginning the restraint stress protocol (Figure 1). As shown in Figure 1, the animals were subject to a tight schedule of behavioral characterization, with a minimal time interval between each test, which could lead to cross-testing interferences. However, the analysis of the performance of control animals in the successive tests did not show evident differences from historic controls where rats of the same age and strain were tested in each different test with wider time gaps between the different tests (Cunha et al., 2006; Cognato et al., 2010; Carmo et al., 2014; Coelho et al., 2014; Matheus et al., 2016). All behavior tests were carried out by two experimenters who were unaware of the phenotypes or drug treatments, in a sound-attenuated room with an eight lux illumination and visual cues on the walls, to which the animals were previously habituated. The apparatuses were cleaned with 20% ethyl alcohol to remove any odors after testing each animal.

Locomotion and exploratory behavior were monitored using an open-field arena made of dark gray PVC measuring 100 × 100 cm^2^ (divided by white lines into 25 squares of 20 × 20 cm^2^) and was surrounded by 40-cm high walls. Each rat was placed in the center of the open field and the following variables were recorded for 10 min: number of peripheral squares (adjacent to the walls) crossed (peripheral locomotion), number of central squares (away from the walls) crossed (central locomotion) and total locomotion (peripheral locomotion plus central locomotion).

Anxiety was further assessed using the elevated plus-maze, which consisted of four arms of the same size (40 cm × 5 cm) arranged in the form of a cross and raised 50 cm above the floor. Two opposed arms were surrounded by 30 cm high opaque black Plexiglas walls, except for the entrance (closed arms) while the other two had no walls (open arms). Each animal was placed on the central square of the maze facing an enclosed arm and was allowed to explore the maze for 5 min. The number of entries and the time spent in both open and closed arms were recorded, considering an entry only when the whole body and four paws were inside an arm.

The depressive-like behavior was evaluated in the forced swimming test, where rats were placed in individual glass cylinders (40 cm in height and 17 cm in diameter) containing water (water depth was 30 cm, kept at 25 ± 1°C) to measure the total duration of immobility, climbing, and swimming during a 10-min session. A rat was regarded as immobile when floating motionless or making only those movements necessary to keep its head above the water. The climbing behavior was defined as upward-directed movements of the forepaws usually along the side of the swimming chamber and the swimming behavior is defined as movement (usually horizontal) throughout the swimming chamber; diving and face shaking behaviors were not considered.

Anhedonic-like behavior was evaluated with the sucrose preference test, where rats were first single-housed in a cage with two bottles and free access to food. After 4 h of habituation, one bottle was randomly switched to contain 1.2% sucrose solution and the total consumption of water and sucrose solution was measured at the end of a 16 h test period (12 h dark phase plus 4 h light phase). Sucrose preference was calculated as the ratio of sucrose vs. total intake.

Spatial memory was evaluated using a 2-trials *Y*-maze paradigm (Dellu et al., 1997). The test was carried out in a Plexiglas apparatus with equal three arms (10 cm wide, 35 cm long, and walls of 25 cm height) in a *Y*-shape, separated by equal angles. The test consists of two sessions of 5 min duration separated by a 2-h inter-trial interval. During the first session, the rat was placed at the end of one arm and allowed to explore the two available arms since the third arm (the novel arm) was blocked by a guillotine door. During the second session, the “‘novel”’ arm was opened and the rat was again placed in the start arm and allowed to explore the three arms. Memory performance was evaluated by measuring the time spent exploring the “novel” arm compared to the exploration of the other two arms. An entry into an arm was defined as the placement of all four paws into the arm.

Hippocampal-dependent memory was also evaluated using the object displacement test, where rats were exposed to two identical objects in the same open field apparatus in which they were habituated and were allowed to explore for 5 min the objects fixed in opposite corners 10 cm away from walls and 70 cm apart from each other. In the test trial, carried out 2-h after, rats were again placed for 5 min in the open field arena, except that one of the objects was moved to a novel position. Memory performance was quantified with an object displacement index defined as the ratio between the time exploring the object in the novel location over the total time exploring both objects. Exploration of an object is defined as directing the nose to the object at a distance equal to or less than 2 cm from the object and/or touching it with the nose; rearing on to the object was not considered exploratory behavior.

The sequence of the tests is indicated in Figure 1.

### mRNA Expression

After completion of the battery of behavior analysis, rats were sacrificed by decapitation under deep anesthesia upon exposure to a halothane-saturated atmosphere. One hippocampus or part of the prefrontal cortex of each rat was used to extract total RNA with a MagNA Lyser Instrument and a MagNA Pure Compact RNA Isolation kit (Roche, Portugal), according to the manufacturer’s instructions. The integrity, quantity, and purity of the RNA yields were checked by electrophoresis and spectrophotometry. Reverse transcription for first-strand cDNA synthesis from each sample was performed using a random hexamer primer with the Transcriptor First Strand cDNA Synthesis kit (Roche), according to the manufacturer’s instructions. The resulting cDNAs were used as templates for real-time PCR, which was carried out on the LightCycler instrument (Roche) using the FastStart DNA Master SYBR Green I kit (Roche). The mRNA expression of the marker of microglia “activation” Iba1 (ionized calcium-binding adaptor molecule 1), of the pro-inflammatory cytokines interleukin-1β (IL1β) and tumor necrosis factor α (TNFα) and of P_2X7_R, was calculated relative to GADPH (glyceraldehyde 3-phosphate dehydrogenase) mRNA expression, using the following primers (from Tib MolBiol, Germany): Iba1 (forward: 5′-TGC GCA AGA GAT CTG CCA TC-3′; reverse: 5′-ACC AGT TGG CTT CTG GTG TT-3′); IL1β (forward: 5′-ATG AGA GCA TCC AGC TTC AAA TC-3′; reverse: 5′-CAC ACT AGC AGG TCG TCA TCA TC-3′); TNFα (forward: 5′-CGA GAT GTG GAA CTG GCA GA-3′; reverse: 5′-CTA CGG GCT TGT CAC TCG A-3′); P2rx7 (forward: 5′-CTG CCT CCC GTC TCA ACT AC-3′; reverse: 5′-GCC TCT CTG GAT AGC ACG AT-3′); GAPDH (forward: 5′-CCC TTC ATT GAC CTC AAC TAC-3′; reverse: 5′-CTT CTC CAT GGT GGT GAA GAC-3′). Quantification was carried out based on standard curves run simultaneously with the test samples generated by conventional PCR amplification, as previously described (Costenla et al., 2011; Rebola et al., 2011). The purity and specificity of the resulting PCR products were assessed by melting curve analysis and electrophoresis. Control reactions were performed to verify that no amplification occurred without cDNA.

### Receptor Binding Assay

The binding assays were performed as previously described (Cunha et al., 2006), using the second hippocampus and the rest of the prefrontal cortex from each rat. After purifying whole membranes by centrifugation-based fractionation (Rebola et al., 2005), the membranes were resuspended in Tris-Mg solution (containing 50 mM Tris and 10 mM MgCl_2_, pH 7.4) with 4 U/ml of adenosine deaminase (to remove endogenous adenosine). Binding with 2 nM of ^3^H-SCH58261 (specific activity of 77 Ci/mmol; prepared by GE Healthcare and offered by E.Ongini, Schering-Plough, Italy), a supramaximal concentration of this selective A_2A_R ligand (Lopes et al., 2004), was performed for 1 h at room temperature with 286–343 (hippocampus) or 54–71 μg of protein (prefrontal cortex), with constant swirling. The binding reactions were stopped by the addition of 4 ml of ice-cold Tris-Mg solution and filtration through Whatman GF/C filters (GE Healthcare). The radioactivity was measured with 2 ml of scintillation liquid (AquaSafe 500 Plus, Zinsser Analytic). The specific binding was expressed as fmol/mg protein and was estimated by subtraction of the non-specific binding, which was measured in the presence of 12 μM of xanthine amine congener (XAC; Sigma), an antagonist of adenosine receptors. All binding assays were performed in duplicate.

### Calcium Transients in N9 Microglial Cells

A murine microglial cell line, N9 (a kind gift from Professor Claudia Verderio, CNR Institute of Neuroscience, Milan, Italy), was grown as previously described (Gomes et al., 2013) in an RPMI medium supplemented with 30 mM glucose (Sigma), 100 U/ml penicillin and 100 μg/ml streptomycin (GIBCO, Invitrogen, Portugal) and maintained at 37°C in an incubator with a humidified atmosphere with 5% CO_2_, until reaching confluence. N9 cells were then detached using 0.05% trypsin (T3924, Sigma) for 5 min, resuspended in RPMI after washing and centrifugation, and counted using a hemocytometer with trypan blue. N9 cells were then seeded in a 48-multiwell at a density of 0.02 × 10^6^ cells and remained in culture for 48 h. Then, cells were incubated for 45 min with Fluo-4-AM (4 μM; Life Technologies) dissolved in recording buffer (132 mM NaCl, 4 mM KCl, 1.4 mM MgCl_2_, 6 mM glucose, 10 mM HEPES, 1.8 mM CaCl_2_; pH 7.4) with 0.05% bovine serum albumin to facilitate probe entry into the cells, as previously described (Simões et al., 2012). The cells were then washed and left in a recording buffer for 15 min to allow complete Fluo-4 AM de-esterification. In some experimental conditions, the following modifiers of the evoked signals were added to the recording buffer during the de-esterification and kept until the end of the experiment: 1 μM JNJ47965567 (2-(phenylthio)-N-[[tetrahydro-4-(4-phenyl-1-piperazinyl)-2H-pyran-4-yl]methyl-3-pyridinecarboxamide, a selective P_2X7_R antagonist from Tocris), 50 nM SCH58261 (2-(2-furanyl)-7-(2-phenylethyl)-7H-pyrazolo[4,3-e][1,2,4]triazolo[1,5-c]pyrimidin-5-amine, a selective A_2A_R antagonist from Tocris) or 100 nM CGS21680 (4-[2-[[6-amino-9-(*N*-ethyl-β-D-ribofuranuronamidosyl)-9H-purin-2-yl]amino]ethyl]benzenepropanoic acid, a selective A_2A_R agonist from Tocris).

After de-esterification, cytosolic Ca^2+^-dependent fluorescence was recorded using a VICTOR^3^ Multiplate reader (Perkin Elmer) with Wallac 1420 software, using an exciting wavelength of 485 nm and recording the emission wavelength at 530 nm, close to the ideal wavelength to monitor Fluo-4 fluorescence (494/506 nm). The baseline fluorescence was recorded at 0.2 Hz. [Ca^2+^]_i_ transients were triggered by the application of different stimuli, either 100 μM BzATP [2′(3′)-O-(4-benzoylbenzoyl)adenosine 5′-triphosphate, a selective P_2X7_R agonist from Sigma], 100 nM CGS21680 or 100 μM glutamate (to mimic excitotoxic conditions, from Sigma) and fluorescence was recorded for 5 min at 0.6 Hz (Janks et al., 2018). When glutamate was used as a trigger of Ca^2+^ transients, experiments were performed with the recording buffer without MgCl_2_ and with 133.4 mM NaCl. After recording the stimulus-induced [Ca^2+^]_i_ transient response, cells were exposed to ionomycin (10 μM, Tocris) to induce a steep increase of extracellular Ca^2+^ influx and consequently a maximum fluorescence response.

The fluorescence data were background-corrected by subtracting the mean fluorescence value of N9 cells that were not incubated with Fluo-4-AM. Intracellular calcium concentration was estimated for each time point using the formula: [Ca^2+^] = K_d_ × (F − F_min_)/(F_max -_ F), in which K_d_ is the dissociation constant of Fluo-4 (345 nM), F is the fluorescence recorded at each time point, F_max_ is the maximal fluorescence, obtained upon ionomycin application, and F_min_ is the minimal fluorescence. The magnitude of [Ca^2+^]_i_ transients evoked by each stimulus (Δ[Ca^2+^]_i_) was obtained subtracting the mean of basal levels from the maximum value after stimulus application.

### Electrophysiological Recordings

Rats were decapitated after anesthesia and the brain was quickly removed and placed in ice-cold, oxygenated (95% O_2_, 5% CO_2_) artificial cerebrospinal fluid (ACSF; in mM: 124.0 NaCl, 4.4 KCl, 1.0 Na_2_HPO_4_, 25.0 NaHCO_3_, 2.0 CaCl_2_, 1.2 MgCl_2_, 10.0 glucose). Using a McIlwain tissue chopper (Brinkmann Instruments, NY, USA), slices (400 μm-thick) from the dorsal hippocampus were cut transverse to its long axis and placed in a holding chamber with oxygenated ACSF. Slices were allowed to recover for at least 1 h before being transferred to a submerged recording chamber and superfused at 3 mL/min with oxygenated ACSF kept at 30.5°C.

Extracellular field excitatory post-synaptic potential (fEPSP) were recorded as previously described (Costenla et al., 2011) with the stimulating bipolar concentric electrode placed in the proximal CA1 *stratum radiatum* for stimulation of the Schaffer collaterals and the recording electrode, filled with 4 M NaCl (2–5 MΩ resistance), placed in the CA1 *stratum radiatum* targeting the distal dendrites of pyramidal neurons. Stimulation was delivered every 20 s with rectangular pulses of 0.1 ms duration using either a Grass S44 or a Grass S48 square pulse stimulator (Grass Technologies, RI, USA). After amplification (ISO-80, World Precision Instruments, Hertfordshire, UK), the recordings were digitized (BNC-2110, National Instruments, Newbury, UK), averaged in groups of three, and analyzed using the WinLTP version 2.10 software (WinLTP Limited, Bristol, UK; Anderson and Collingridge, 2007). The intensity of stimulation was chosen between 30–50% of maximal fEPSP response, determined based on input/output curves in which the fEPSP slope was plotted vs. stimulus intensity. Alterations of basal synaptic transmission were quantified as the percentage change of the average value of the fEPSP slope taken from 15–20 min after beginning exposure to the tested drug applied through the superfusion medium, relative to the average value of the fEPSP slope during the 5 min that preceded the application of each modifying drug. Long-term potentiation (LTP) was induced by a high-frequency stimulation (HFS) train (100 Hz for 1 s). LTP was quantified as the percentage change of the average fEPSP slope taken between 55 and 60 min after LTP induction relative to the average slope of the fEPSP measured during the 10 min that preceded LTP induction. The effect of drugs on LTP was assessed by comparing LTP magnitude in the absence and presence of the drug in experiments carried out in different slices from the same animal.

### Statistics

Data are presented as the mean ± SEM of *n* experiments (i.e., *n* independent rats or cell cultures). The comparison of control and stressed rats and the effect of drugs was analyzed using a two-tailed unpaired Student’s *t*-test. When testing the impact of a drug on the effects of stress, the data were first analyzed with a two-way ANOVA followed by a Newman–Keuls *post hoc* test. The comparison between the effect of multiple drugs was carried out using a Dunnett’s test. All tests were performed using Prism 6.0 software (GraphPad, San Diego, CA, USA) considering significance at a 95% confidence interval.

## Results

**Figure 2:**
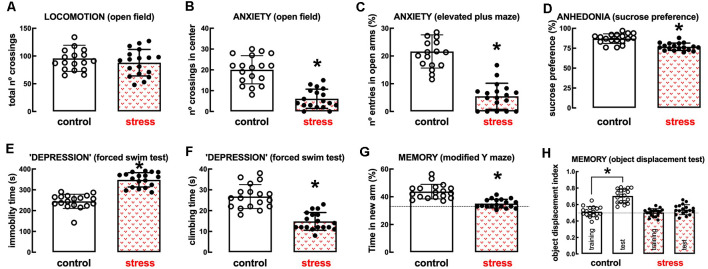
Male adult Wistar rats (8–10 weeks old) subject to a protocol of restraint stress (4 h/day) during 14 days display the expected features of depressed rats. Compared with non-stressed control rats (open bars), stressed rats (red checkered bars) displayed a preserved locomotor activity as evaluated in the open field **(A)**, anxiety-like behavior as evaluated in the open field **(B)**, and in the elevated plus-maze **(C)** tests, anhedonia as evaluated in the sucrose preference test **(D)**, helpless-like behavior as evaluated by the forced-swimming test **(E,F)** and impaired memory performance as evaluated by a modified Y maze test **(G)** and an object-displacement test **(H)**. Data are shown as mean ± SEM; *n* = 16–18 rats per group. *P < 0.001 using a Student’s *t*-test.

The model of repeated restraint stress triggers robust and reproducible behavioral alterations of mood and memory in adult rats (see Figure 2). Thus, whereas there was no significant change of spontaneous locomotion (*n* = 18; *t* = 0.991, *p* = 0.328, unpaired Student’s *t*-test; Figure 2A), stressed rats displayed a thigmotaxic behavior indicative of an increased anxiety-like profile, as indicated by the decreased number of crossings in the center of the open field (*n* = 18; *t* = 7.229, *p* < 0.001, unpaired Student’s *t*-test; Figure 2B). This was confirmed in the elevated plus-maze where stressed rats displayed a decreased number of entries in the open arms (*n* = 18; *t* = 9.002, *p* < 0.001, unpaired Student’s *t*-test; Figure 2C) and decreased time in the open arms (*n* = 18; *t* = 8.628, *p* < 0.001, unpaired Student’s *t*-test). Stressed rats also displayed anhedonic behavior in a sucrose preference test (*n* = 18; *t* = 5.673, *p* < 0.001, unpaired Student’s *t*-test; Figure 2D) and an increased immobility time in the forced swimming test (*n* = 18; *t* = 9.959, *p* < 0.001, unpaired Student’s *t*-test; Figure 2E), as well as a decreased time spent climbing the walls of the swimming container (*n* = 18; *t* = 7.069, *p* < 0.001, unpaired Student’s *t*-test; Figure 2F), indicative of depressive-like behavior. Short-term memory was also deteriorated in stressed compared to control rats, as observed by a decreased time searching the novel (previously hidden) arm of a *Y*-maze (*n* = 18; *t* = 6.033, *p* < 0.001, unpaired Student’s *t*-test; Figure 2G) and a decreased preference to explore the displaced object (*t* = 8.009, *p* < 0.001 between displaced and non-displaced object in control rats and *t* = 1.885, *p* = 0.069 between displaced and non-displaced object in stressed rats, unpaired Student’s *t*-test; Figure 2H).

**Figure 3:**
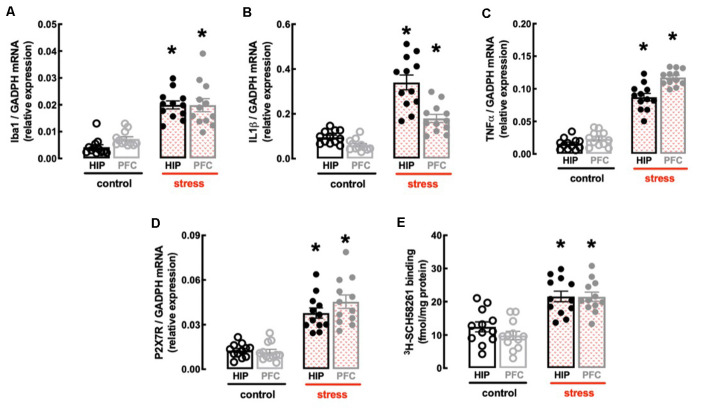
Male adult Wistar rats (8–10 weeks old) subject to a protocol of restraint stress (4 h/day) during 14 days display an increased expression of inflammatory markers and an up-regulation of P_2X7_ and A_2A_ receptors in the hippocampus (black) and prefrontal cortex (gray). Compared with non-stressed control rats (open bars), stressed rats (red checkered bars) displayed an increased expression of the microglia marker Iba1 **(A)**, of interleukin 1β (IL1β; **B**), of tumor necrosis factor α (TNFα; **C**), and P_2X7_ receptors (P_2X7_R; **D**) as well as an increased density of A_2A_ receptors (A_2A_R; **E**) as assessed by the binding density of a supramaximal concentration of the selective A_2A_R antagonist ^3^H-SCH58261 (2 nM). Data are shown as mean ± SEM; *n* = 11–12 rats per group. *P < 0.001 vs. control using a Student’s *t*-test.

In line with the involvement of the hippocampus and prefrontal cortex in processing mood and memory-related information (de Kloet et al., 2005) and the association of a heightened inflammatory status in these brain regions in mood impaired animals (Troubat et al., 2021), repeated restraint stress increased the expression of inflammatory markers in the hippocampus and prefrontal cortex (Figures 3A–C). Thus, the hippocampus of stressed rats displayed increased mRNA levels of the marker of microglia “activation” Iba1 (*n* = 12; *t* = 9.095, *p* < 0.001, unpaired Student’s *t*-test; Figure 3A) and of the pro-inflammatory cytokines interleukin-1β (IL1β; *n* = 12; *t* = 7.194, *p* < 0.001, unpaired Student’s *t*-test; Figure 3B) and tumor necrosis factor α (TNFα; *n* = 12; *t* = 11.46, *p* < 0.001, unpaired Student’s *t*-test; Figure 3C). Likewise, the prefrontal cortex of stressed rats also displayed increased mRNA levels of Iba1 (*n* = 12; *t* = 4.928, *p* < 0.001, unpaired Student’s *t*-test; Figure 3A), IL1β (*n* = 12; *t* = 6.028, *p* < 0.001, unpaired Student’s *t*-test; Figure 3B) and TNFα (*n* = 12; *t* = 20.01, *p* < 0.001, unpaired Student’s *t*-test; Figure 3C).

Finally, the protocol of restraint stress triggered an up-regulation of P_2X7_R and of A_2A_R (Figures 3D,E), two purinergic receptor systems that have been implicated in mood alterations caused by stressful conditions (e.g., Kaster et al., 2015; Iwata et al., 2016). Thus, stressed rats displayed an increased expression of P_2X7_R mRNA in the hippocampus (*n* = 12; *t* = 6.82, *p* < 0.001, unpaired Student’s *t*-test; Figure 3D) and prefrontal cortex (*n* = 12; *t* = 6.967, *p* < 0.001, unpaired Student’s *t-*test; Figure 3D), as well as an increased binding density of the selective A_2A_R antagonist ^3^H-SCH58261 in the hippocampus (*n* = 12; *t* = 4.212, *p* < 0.001, unpaired Student’s *t*-test; Figure 3E) and prefrontal cortex (*n* = 12; *t* = 6.181, *p* < 0.001, unpaired Student’s *t*-test; Figure 3E).

### Impact of the P_2X7_R Antagonist BBG

**Figure 4:**
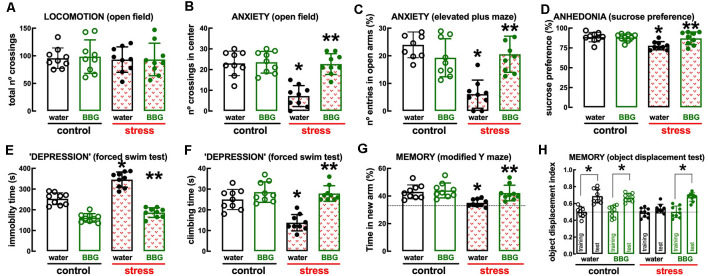
Male adult Wistar rats (8–10 weeks old) subject to a protocol of restraint stress (4 h/day) during 14 days display the expected features of depressed rats, which were prevented by the P_2X7_ receptor antagonist Brillant Blue G (BBG). Whereas BBG treatment (45 mg/kg, ip, daily, beginning 3 days before the stress protocol and until the sacrifice of the animals; green) was devoid of effects in non-stressed control rats (open bars), BBG prevented all behavioral modifications of stressed rats (red checkered bars): without modification of locomotor activity as evaluated in the open field **(A)**, BBG prevented anxiety-like behavior as evaluated in the open field **(B)** and in the elevated plus-maze **(C)** tests, anhedonia as evaluated in the sucrose preference test **(D)**, helpless-like behavior as evaluated by the forced-swimming test **(E,F)** and impaired memory performance as evaluated by a modified Y maze test **(G)** and an object-displacement test **(H)**. Data are shown as mean ± SEM; *n* = 8–9 rats per group. *P < 0.05 vs. control-water, **P < 0.05 vs. stress-water using a Tukey’s multiple comparisons *post hoc* test after a two-way ANOVA.

**Figure 5:**
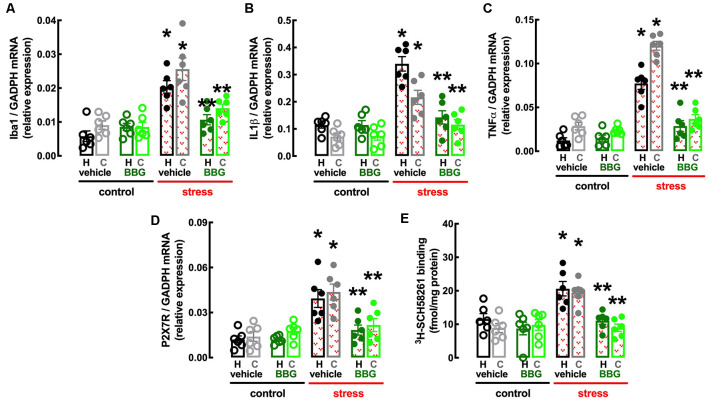
Male adult Wistar rats (8–10 weeks old) subject to a protocol of restraint stress (4 h/day) during 14 days display an increased expression of inflammatory markers and an up-regulation of P_2X7_ and A_2A_ receptors in the hippocampus (black, dark green) and prefrontal cortex (gray, light green) which were prevented by the P_2X7_ receptor antagonist Brillant Blue G (BBG). Whereas BBG treatment (45 mg/kg, ip, daily, beginning 3 days before the stress protocol and until the sacrifice of the animals; green) was devoid of effects in non-stressed control rats (open bars), BBG prevented all alterations of stressed rats (red checkered bars), namely the increased expression of the microglia marker Iba1 **(A)**, of interleukin 1β (IL1β; **B**), of tumor necrosis factor α (TNFα; **C**), and P_2X7_ receptors (P_2X7_R; **D**) as well as an increased density of A_2A_ receptors (A_2A_R; **E**) as assessed by the binding density of a supramaximal concentration of the selective A_2A_R antagonist ^3^H-SCH58261 (2 nM). Data are shown as mean ± SEM; *n* = 5–7 rats per group. *P < 0.05 vs. control-water, **P < 0.05 vs. stress-water using a Tukey’s multiple comparisons *post hoc* test after a two-way ANOVA.

The P_2X7_R-prefering antagonist Brillant Blue G (BBG, 45 mg/kg) was devoid of effects in control rats but attenuated or prevented the behavioral and neurochemical alterations caused by repeated stress (Figures 4, 5). Thus, BBG prevented the stress-induced decrease of the number of crossings in the central area of the open field (effect of stress *F*_(1,32)_ = 22.13, *p* < 0.001; effect of BBG *F*_(1,32)_ = 20.41, *p* < 0.001; interaction *F*_(1,32)_ = 17.66, *p* < 0.001; two-way ANOVA; Figure 4B), the stress-induced decrease of the number of entries in the open arms of the elevated plus maze (effect of stress *F*_(1,32)_ = 17.96, *p* < 0.001; effect of BBG *F*_(1,32)_ = 6.248, *p* = 0.018; interaction *F*_(1,32)_ = 23.89, *p* < 0.001; two-way ANOVA; Figure 4C), the stress-induced decrease of the time spent in the open arms of the elevated plus maze (effect of stress *F*_(1,32)_ = 23.28, *p* < 0.001; effect of BBG *F*_(1,32)_ = 4.187, *p* = 0.044; interaction *F*_(1,32)_ = 25.61, *p* < 0.001; two-way ANOVA), the stress-induced decrease of sucrose preference (effect of stress *F*_(1,32)_ = 8.737, *p* = 0.006; effect of BBG *F*_(1,32)_ = 4.753, *p* = 0.037; interaction *F*_(1,32)_ = 6.044, *p* = 0.019; two-way ANOVA; Figure 4D), the stress-induced increase of immobility in the forced swimming test (effect of stress *F*_(1,32)_ = 39.91, *p* < 0.001; effect of BBG *F*_(1,32)_ = 181.4, *p* < 0.001; interaction *F*_(1,32)_ = 13.02, *p* = 0.001; two-way ANOVA; Figure 4E), the stress-induced decrease of the time climbing the wall in the forced swimming test (effect of stress *F*_(1,32)_ = 16.35, *p* < 0.001; effect of BBG *F*_(1,32)_ = 36.11, *p* < 0.001; interaction *F*_(1,32)_ = 12.87, *p* = 0.001; two-way ANOVA; Figure 4F), the stress-induced decrease of the time spent in the novel arm of the *Y*-maze (effect of stress *F*_(1,32)_ = 9.243, *p* = 0.005; effect of BBG *F*_(1,32)_ = 6.434, *p* = 0.016; interaction *F*_(1,32)_ = 3.596, *p* = 0.067; two-way ANOVA; Figure 4G), and the stress-induced decrease of the relative time exploring the displaced object (*t* = 1.928, *p* = 0.072 between displaced and non-displaced object in stressed rats treated with vehicle and *t* = 6.246, *p* < 0.001 between displaced and non-displaced object in stressed rats treated with BBG, unpaired Student’s *t*-test; Figure 4H).

BBG also attenuated the stress-induced increase in the expression of the marker of “activated” microglia Iba1 in the hippocampus (effect of stress *F*_(1,20)_ = 30.51, *p* < 0.001; effect of BBG *F*_(1,20)_ = 5.295, *p* = 0.032; interaction *F*_(1,20)_ = 16.96, *p* = 0.001; two-way ANOVA; Figure 5A) and prefrontal cortex (effect of stress *F*_(1,20)_ = 30.52, *p* < 0.001; effect of BBG *F*_(1,20)_ = 9.150, *p* = 0.007; interaction *F*_(1,20)_ = 7.524, *p* = 0.012; two-way ANOVA; Figure 5A), as well as in the levels of mRNA of both IL1β in the hippocampus (effect of stress *F*_(1,20)_ = 38.13, *p* < 0.001; effect of BBG *F*_(1,20)_ = 23.05, *p* < 0.001; interaction *F*_(1,20)_ = 23.15, *p* < 0.001; two-way ANOVA; Figure 5B) and prefrontal cortex (effect of stress *F*_(1,20)_ = 24.39, *p* < 0.001; effect of BBG *F*_(1,20)_ = 6.641, *p* = 0.018; interaction *F*_(1,20)_ = 7.505, *p* = 0.013; two-way ANOVA; Figure 5B) and of TNFα in the hippocampus (effect of stress *F*_(1,20)_ = 59.05, *p* < 0.001; effect of BBG *F*_(1,20)_ = 19.99, *p* < 0.001; interaction *F*_(1,20)_ = 24.96, *p* < 0.001; two-way ANOVA; Figure 5C) and prefrontal cortex (effect of stress *F*_(1,20)_ = 152.8, *p* < 0.001; effect of BBG *F*_(1,20)_ = 108.7, *p* < 0.001; interaction *F*_(1,20)_ = 85.38, *p* < 0.001; two-way ANOVA; Figure 5C).

The treatment with BBG also attenuated the stress-induced up-regulation of P_2X7_R in the hippocampus (effect of stress *F*_(1,20)_ = 21.31, *p* < 0.001; effect of BBG *F*_(1,20)_ = 8.316, *p* = 0.009; interaction *F*_(1,20)_ = 8.222, *p* = 0.009; two-way ANOVA; Figure 5D) and prefrontal cortex (effect of stress *F*_(1,20)_ = 18.12, *p* < 0.001; effect of BBG *F*_(1,20)_ = 5.305, *p* = 0.032; interaction *F*_(1,20)_ = 10.52, *p* < 0.001; two-way ANOVA; Figure 5D). Remarkably, BBG also attenuated the stress-induced up-regulation of A_2A_R in the hippocampus (effect of stress *F*_(1,20)_ = 10.85, *p* = 0.004; effect of BBG *F*_(1,20)_ = 13.01, *p* = 0.002; interaction *F*_(1,20)_ = 3.766, *p* = 0.067; two-way ANOVA; Figure 5E) and prefrontal cortex (effect of stress *F*_(1,20)_ = 12.21, *p* = 0.002; effect of BBG *F*_(1,20)_ = 11.84, *p* = 0.003; interaction *F*_(1,20)_ = 16.96, *p* = 0.001; two-way ANOVA; Figure 5E).

### Impact of the Adenosine Receptor Antagonist Caffeine

**Figure 6:**
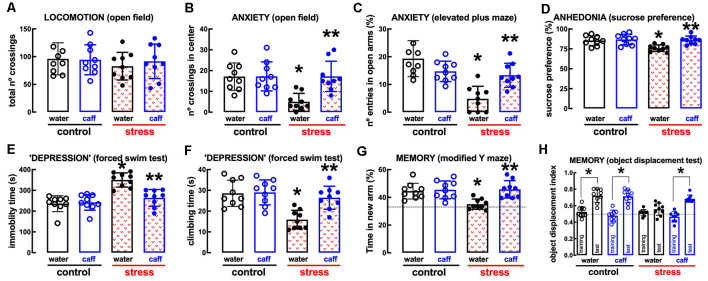
Male adult Wistar rats (8–10 weeks old) subject to a protocol of restraint stress (4 h/day) during 14 days display the expected features of depressed rats, which were prevented by the adenosine receptor antagonist caffeine (caff). Whereas caffeine consumption (0.3 g/L, po, beginning 3 days before the stress protocol and until the sacrifice of the animals; blue) was devoid of effects in non-stressed control rats (open bars), caffeine prevented all behavioral modifications of stressed rats (red checkered bars): without modification of locomotor activity as evaluated in the open field **(A)**, caffeine prevented anxiety-like behavior as evaluated in the open field **(B)** and in the elevated plus-maze **(C)** tests, anhedonia as evaluated in the sucrose preference test **(D)**, helpless-like behavior as evaluated by the forced-swimming test **(E,F)** and impaired memory performance as evaluated by a modified Y maze test **(G)** and an object-displacement test **(H)**. Data are shown as mean ± SEM; *n* = 8–9 rats per group. *P < 0.05 vs. control-water, **P < 0.05 vs. stress-water using a Tukey’s multiple comparisons *post hoc* test after a two-way ANOVA.

**Figure 7:**
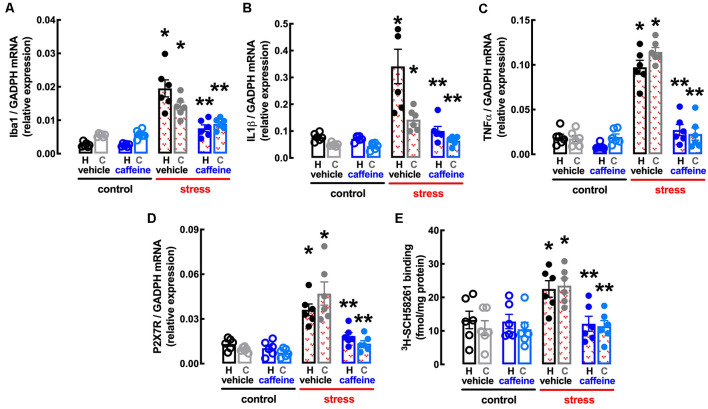
Male adult Wistar rats (8–10 weeks old) subject to a protocol of restraint stress (4 h/day) during 14 days display an increased expression of inflammatory markers and an up-regulation of P_2X7_ and of A_2A_ receptors in the hippocampus (black, dark blue) and prefrontal cortex (gray, light blue) which were prevented by the adenosine antagonist caffeine (caff). Whereas caffeine consumption (0.3 g/L, po, beginning 3 days before the stress protocol and until the sacrifice of the animals; blue) was devoid of effects in non-stressed control rats (open bars), caffeine prevented all alterations of stressed rats (red checkered bars), namely the increased expression of the microglia marker Iba1 **(A)**, of interleukin 1β (IL1β; **B**), of tumor necrosis factor α (TNFα; **C**) and P_2X7_ receptors (P_2X7_R; **D**) as well as an increased density of A_2A_ receptors (A_2A_R; **E**) as assessed by the binding density of a supramaximal concentration of the selective A_2A_R antagonist ^3^H-SCH58261 (2 nM). Data are shown as mean ± SEM; *n* = 5–7 rats per group. *P < 0.05 vs. control-water, **P < 0.05 vs. stress-water using a Tukey’s multiple comparisons *post hoc* test after a two-way ANOVA.

The non-selective adenosine receptor antagonist, caffeine (0.3 g/L, *p.o*.), which affords neuroprotection through the antagonism of A_2A_R (e.g., Dall’Igna et al., 2007; Cognato et al., 2010; Kaster et al., 2015), was devoid of effects in control rats but attenuated or prevented the behavioral and neurochemical alterations caused by repeated stress (Figures 6, 7). Thus, caffeine prevented the stress-induced decrease of the number of crossing in the central area of the open field (effect of stress *F*_(1,32)_ = 8.160, *p* = 0.007; effect of caffeine *F*_(1,32)_ = 8.459, *p* = 0.007; interaction *F*_(1,32)_ = 8.160, *p* = 0.007; two-way ANOVA; Figure 6B), the stress-induced decrease of the number of entries in the open arms of the elevated plus maze (effect of stress *F*_(1,32)_ = 23.91, *p* < 0.001; effect of caffeine *F*_(1,32)_ = 1.463, *p* = 0.235; interaction *F*_(1,32)_ = 16.27, *p* < 0.001; two-way ANOVA; Figure 6C), the stress-induced decrease of sucrose preference (effect of stress *F*_1,64_ = 15.96, *p* < 0.001; effect of caffeine *F*_3, 64_ = 6.544, *p* = 0.001; interaction *F*_3, 64_ = 3.828, *p* = 0.014; two-way ANOVA; Figure 6D), the stress-induced increase of immobility in the forced swimming test (effect of stress *F*_(1,32)_ = 29.31, *p* < 0.001; effect of caffeine *F*_(1,32)_ = 10.13, *p* = 0.003; interaction *F*_(1,32)_ = 13.58, *p* = 0.001; two-way ANOVA; Figure 6E), the stress-induced decrease of the time climbing the wall in the forced swimming test (effect of stress *F*_(1,32)_ = 16.45, *p* < 0.001; effect of caffeine *F*_(1,32)_ = 8.564, *p* = 0.006; interaction *F*_(1,32)_ = 7.247, *p* = 0.001; two-way ANOVA; Figure 6F), the stress-induced decrease of the time spent in the novel arm of the *Y*-maze (effect of stress *F*_(1,32)_ = 5.879, *p* = 0.021; effect of caffeine *F*_(1,32)_ = 9.671, *p* = 0.004; interaction *F*_(1,32)_ = 6.851, *p* = 0.013; two-way ANOVA; Figure 6G), and the stress-induced decrease of the relative time exploring the displaced object (*t* = 1.492, *p* = 0.161 between displaced and non-displaced object in stress rats treated with vehicle and *t* = 8.637, *p* < 0.001 between displaced and non-displaced object in stress rats treated with caffeine, unpaired Student’s *t*-test; Figure 6H).

Caffeine also attenuated the stress-induced increase in the expression of the marker of “activated” microglia Iba1 in the hippocampus (effect of stress *F*_(1,20)_ = 63.06, *p* < 0.001; effect of caffeine *F*_(1,20)_ = 19.07, *p* < 0.001; interaction *F*_(1,20)_ = 18.35, *p* < 0.001; two-way ANOVA; Figure 7A) and prefrontal cortex (effect of stress *F*_(1,20)_ = 57.53, *p* < 0.001; effect of caffeine *F*_(1,20)_ = 10.02, *p* = 0.005; interaction *F*_(1,20)_ = 12.92, *p* = 0.002; two-way ANOVA; Figure 7A), as well as in the levels of mRNA of both IL1β in the hippocampus (effect of stress *F*_(1,20)_ = 19.2, *p* < 0.001; effect of caffeine *F*_(1,20)_ = 13.58, *p* = 0.001; interaction *F*_(1,20)_ = 12.34, *p* = 0.002; two-way ANOVA; Figure 7B) and prefrontal cortex (effect of stress *F*_(1,20)_ = 22.15, *p* < 0.001; effect of caffeine *F*_(1,20)_ = 9.351, *p* = 0.006; interaction *F*_(1,20)_ = 30.24, *p* < 0.001; two-way ANOVA; Figure 7B) and of TNFα in the hippocampus (effect of stress *F*_(1,20)_ = 82.54, *p* < 0.001; effect of caffeine *F*_(1,20)_ = 57.24, *p* < 0.001; interaction *F*_(1,20)_ = 31.66, *p* < 0.001; two-way ANOVA; Figure 7C) and prefrontal cortex (effect of stress *F*_(1,20)_ = 106.0, *p* < 0.001; effect of caffeine *F*_(1,20)_ = 85.53, *p* < 0.001; interaction *F*_(1,20)_ = 92.56, *p* < 0.001; two-way ANOVA; Figure 7C).

The treatment with caffeine also attenuated the stress-induced up-regulation of P_2X7_R in the hippocampus (effect of stress *F*_(1,20)_ = 35.32, *p* < 0.001; effect of caffeine *F*_(1,20)_ = 15.30, *p* = 0.001; interaction *F*_(1,20)_ = 8.046, *p* = 0.010; two-way ANOVA; Figure 7D) and prefrontal cortex (effect of stress *F*_(1,20)_ = 5.011, *p* = 0.048; effect of caffeine *F*_(1,20)_ = 0.569, *p* = 0.094; interaction *F*_(1,20)_ = 51.4, *p* < 0.001; two-way ANOVA; Figure 7D), as well as the stress-induced up-regulation of A_2A_R in the hippocampus (effect of stress *F*_(1,20)_ = 4.282, *p* = 0.045; effect of caffeine *F*_(1,20)_ = 5.256, *p* = 0.033; interaction *F*_(1,20)_ = 4.369, *p* = 0.050; two-way ANOVA; Figure 7E) and prefrontal cortex (effect of stress *F*_(1,20)_ = 10.98, *p* = 0.004; effect of caffeine *F*_(1,20)_ = 9.302, *p* = 0.006; interaction *F*_(1,20)_ = 8.317, *p* = 0.009; two-way ANOVA; Figure 7E).

### P_2X7_R –A_2A_R Interaction in Microglial N9 Cells

Since we observed crosstalk between BBG and caffeine upon restraint stress, whereby BBG controlled the up-regulation of A_2A_R and caffeine controlled the upregulation of P_2X7_R expression, and the stress-induced behavioral modifications were accompanied by a parallel control of markers of microglia “activation” and neuroinflammation, we next used a microglial N9 cell line to directly investigate a putative crosstalk between P_2X7_R and A_2A_R, since both receptors are present and functional in this microglia cell model (e.g., Ferrari et al., 1996; Gomes et al., 2013).

**Figure 8:**
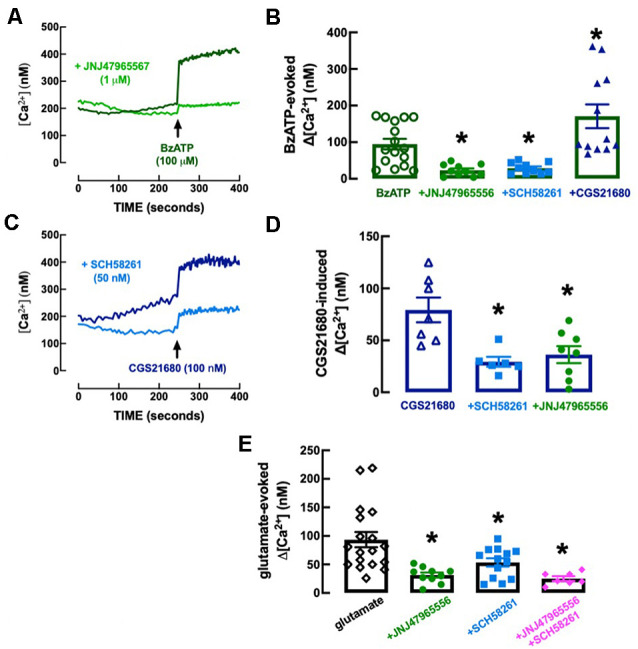
Functional interaction between P_2X7_ and A_2A_ receptors in the control of calcium responses in N9 microglial cell lines. **(A)** The Fluo-4 fluorescence signal reporting alteration of intracellular free calcium levels ([Ca^2+^]_i_) was increased by the P_2X7_ receptor agonist BzATP (100 μM), an effect abolished by the selective P_2X7_ receptor antagonist JNJ47965567 (1 μM), added 15 min before BzATP. **(B)** Furthermore, the addition 15 min before BZATP of the A_2A_ receptor antagonist SCH58261 (50 nM) decreased and the A_2A_ receptor agonist CGS21680 (100 nM) increased BzATP-induced increase of [Ca^2+^]_i_ (Δ[Ca^2+^]_i_). **(C)** CGS21680 also increased [Ca^2+^]_i_ in a manner attenuated by SCH58261, as well as by JNJ47965567 **(D)**, each added 15 min before CGS21680. **(E)** Glutamate (100 μM) also increased [Ca^2+^]_i_, an effect attenuated by both JNJ47965567 and by SCH58261, and their simultaneous presence caused an inhibition similar to each antagonist alone (antagonists being added 15 min before glutamate). The time course recordings are from representative experiments, whereas the bar graphs correspond to *n* = 6–18 independent cultures of N9 microglial cells. *p < 0.05 one-way ANOVA followed by a Dunnett’s *post hoc* test compared to the first bar from the left (stimulus only, without modifiers, which were added 5 min before the stimulus).

The P_2X7_R-preferring agonist BzATP (100 μM) evoked an elevation of intracellular free Ca^2+^ levels (Δ[Ca^2+^]_i_) of 94.8 ± 14.5 nM (*n* = 16), which was inhibited (−76.51 ± 20.02%, *n* = 10–16, *F*_(3, 44)_ = 13.21, *p* = 0.029) in the presence of the selective P_2X7_R antagonist, JNJ4796556 (1 μM), added 15 min before BzATP (Figures 8A,B). The selective A_2A_R agonist CGS21680 (100 nM) also evoked a Δ[Ca^2+^]_i_ of 79.3 ± 11.9 nM (*n* = 7), which was inhibited (−63.0 ± 14.0%, *n* = 6, *F*_(2, 18)_ = 8.67, *p* = 0.003) in the presence of the selective A_2A_R antagonist, SCH58261 (50 nM), added 15 min before CGS21680 (Figures 8C,D). Notably, the Δ[Ca^2+^]_i_ evoked by BzATP (100 μM) was inhibited (−69.5 ± 15.7%, *n* = 10–16, *F*_(3, 44)_ = 12.13, *p* = 0.048) by SCH58261 (50 nM) and potentiated (+80.3 ± 19.5%, *n* = 12–16, *F*_(3, 44)_ = 12.13, *p* = 0.012) by CGS21680 (100 nM; Figure 8B), whereas the Δ[Ca^2+^]_i_ triggered by CGS21680 (100 nM) was inhibited (−54.3 ± 14.7%, *n* = 8, *F*_(2, 18)_ = 8.67, *p* = 0.005) by JNJ47965567 (1 μM; Figure 8D), indicating a crosstalk between P_2X7_R and A_2A_R in the control of Δ[Ca^2+^]_i_ responses in microglial N9 cells. This P_2X7_R-A_2A_R crosstalk is further reinforced by the observation that neither JNJ47965567 (1 μM) nor SCH58216 (50 nM) affected basal [Ca^2+^]_i_ levels (control, no drugs: 284.2 ± 30.2 nM, *n* = 10; 1 μM JNJ47965567: 224.2 ± 32.0 nM, *n* = 9; *F*_3, 34_ = 1.92, *p* = 0.488 vs. control; 50 nM SCH58261: 219.1 ± 17.6 nM, *n* = 8, *F*_3, 34_ = 1.92, *p* = 0.449 vs. control), indicating a lack of tonic P_2X7_R- or A_2A_R-mediated control of Δ[Ca^2+^]_i_ that could hinder the interpretation of the cross-inhibition between both purinergic receptor systems.

We next explored if there was a control by P_2X7_R and by A_2A_R and a crosstalk between both receptors in the control of Δ[Ca^2+^]_i_ evoked by glutamate to mimic a condition of excitotoxicity-induced “activation” of microglia (reviewed in Zhang et al., 2020), irrespective of the receptors involved. Glutamate (100 mM) triggered a Δ[Ca^2+^]_i_ of 93.3 ± 13.4 nM (*n* = 18), which was inhibited either by 1 μM JNJ47965567 (−66.4 ± 13.3%, *n* = 10–18, *F*_4,53_ = 13.56, *p* = 0.002) or by 50 nM SCH58261 (−42.67 ± 8.41%, *n* = 13–18, *F*_4,53_ = 13.56, *p* = 0.050), each added 15 min before BzATP (Figure 8E). Notably, glutamate-induced Δ[Ca^2+^]_i_ was 25.1 ± 4.1 nM (*n* = 7) in the simultaneous presence of JNJ47965567 (1 μM) and SCH58261 (50 nM) indicating an inhibition of −73.1 ± 15.8% (Figure 8E), which was similar to that caused by JNJ47965567 alone (*t* = 0.997, *p* = 0.334).

### P_2X7_R –A_2A_R Interaction in the Control of Hippocampal Synaptic Plasticity

Since we and others have collected evidence for a role of synaptic dysfunction underlying stress-associated behavioral alterations (Duman and Aghajanian, 2012; Kaster et al., 2015) and suggestions of P_2X7_R-mediated synaptic dysfunction add-up to the well-established ability of A_2A_R to control synaptic function (reviewed in Cunha, 2016), we next investigated if P_2X7_R and A_2A_R might interact in the control of synaptic plasticity in excitatory synapses of the dorsal hippocampus.

**Figure 9:**
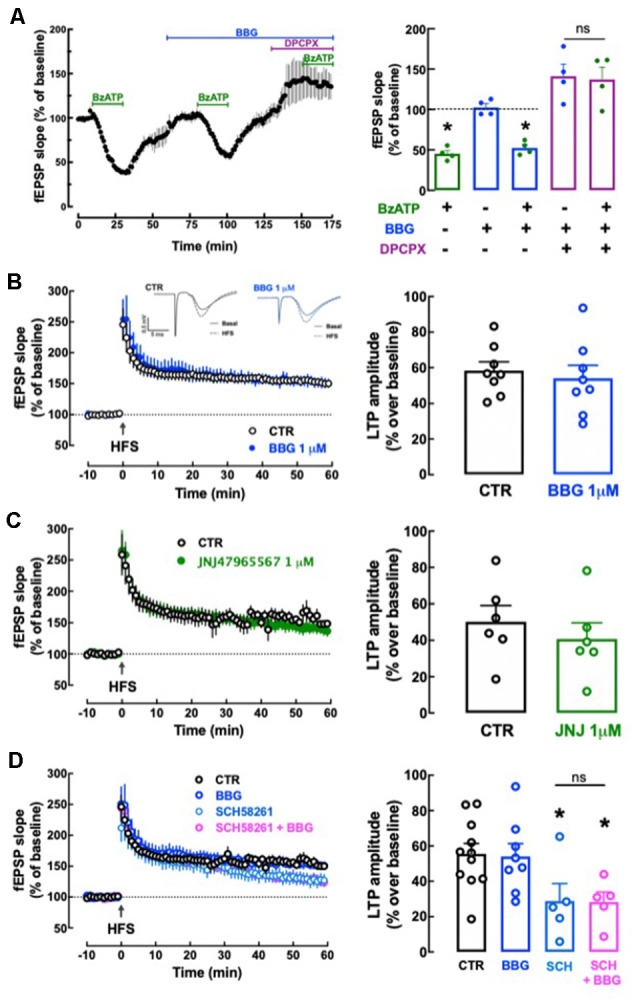
Lack of direct effects of P_2X7_ receptors on hippocampal synaptic plasticity or its modulation by A_2A_ receptors. **(A)** The P_2X7_R agonist BzATP (30 μM) decreased synaptic transmission in Schaffer collaterals-CA1 pyramid synapses of hippocampal slices from adult rats (10–12 weeks old), but this effect was likely mediated through A_1_R since it was prevented by the A_1_R antagonist DPCPX (50 nM) but not by the P_2X7_R antagonist BBG (1 μM). Data are shown as mean ± SEM of *n* = 4; *p < 0.05 vs. control (100%, dashed line). **(B–D)** The P_2X7_R antagonists BBG (1 μM) or JNJ47965567 (JNJ, 1 μM) did not significantly modify the magnitude of Long-term potentiation (LTP; change in field excitatory post-synaptic potential (fEPSP) slope at 50–60 min) induced by a high-frequency stimulation (HFS) train concerning pre-HFS values **(B,C)** and also failed to alter the inhibition of LTP magnitude caused by the A_2A_R antagonist SCH58261 (SCH, 50 nM; **D**). The inserts show recordings obtained in representative experiments of fEPSP responses obtained before (filled line) and 50–60 min after (dotted line) LTP induction in the presence or in the absence (control) of BBG; each trace comprises the stimulus artifact, followed by the presynaptic volley and the fEPSP. All values are shown as mean ± SEM of 5–8 experiments; *p < 0.05 vs. LTP magnitude in the absence of drugs (control). ns: non-significant.

We first tested the effect of P_2X7_R agonist BzATP on basal synaptic transmission. BzATP (30 μM) decreased hippocampal synaptic transmission by 54.75 ± 3.96% (*n* = 4); this effect recovered fully upon washout of BzATP and repeated administrations of 30 μM BzATP caused a similar depression of synaptic transmission (*p* > 0.05). This allowed exploring the pharmacology of BzATP (30 μM)-induced decreased hippocampal synaptic transmission: this effect was unaffected in the presence of 1 μM BBG (−48.98 ± 4.96%, *n* = 4, *t* = 1.245, *p* = 0.260 vs. the effect of BzATP alone) and was fully prevented in the presence of the adenosine A_1_ receptor antagonist, DPCPX (50 nM; 2.97 ± 17.91% alteration of fEPSP slope, *n* = 4; *t* = 3.319, *p* = 0.016 vs. the effect of BzATP alone; Figure 9A). This shows the inexistence of a P_2X7_R-mediated effect (lack of effect of BBG) and indicates that BzATP is rapidly converted by ectonucleotidases (Cunha et al., 1998) into an adenosine analog to indirectly alter hippocampal synaptic transmission through inhibitory A_1_ adenosine receptors (prevention by DPCPX), as previously proposed (Kukley et al., 2004). This precludes the use of BzATP to search for P_2X7_R-mediated effects in hippocampal slices. Instead, we tested the impact of P_2X7_R antagonists on high-frequency induced LTP in Schaffer collaterals-CA1 pyramidal cell synapses. LTP magnitude was not significantly altered by either 1 μM BBG (*n* = 8, *t* = 0.493, *p* = 0.630 vs. LTP magnitude in control conditions, i.e., in the absence of tested drugs) or 1 μM JNJ47965567 (*n* = 6; *t* = 0.754, *p* = 0.468 vs. control LTP magnitude; Figures 9B,C). This does not support a role of P_2X7_R in the control of synaptic plasticity.

We next investigated if P_2X7_R might instead control the known ability of A_2A_R to control hippocampal synaptic plasticity (e.g., Costenla et al., 2011; Lopes et al., 2019). SCH58261 (50 nM) decreased LTP magnitude by −48.10 ± 10.77% (*n* = 5; *t* = 2.440, *p* = 0.029 vs. control LTP magnitude; Figure 9D) and a non-significantly different inhibition of −49.11 ± 7.21% (*n* = 5; *t* = 0.049, *p* = 0.962 vs. LTP magnitude in the SCH58261 alone) was observed in the simultaneous presence of BBG (1 μM) and SCH58261 (50 nM; Figure 9D).

## Discussion

The present study provides compelling novel evidence for a hitherto unrecognized interaction between P_2X7_R and A_2A_R in the control of brain dysfunction. This conclusion is based on the parallel effects of BBG, a P_2X7_R preferring antagonist, and of caffeine, which antagonizes A_2A_R, to prevent neuroinflammation and behavioral alterations upon repeated restraint stress and on the ability of caffeine to prevent P_2X7_R upregulation and of BBG to prevent A_2A_R up-regulation; although these *in vivo* evidence are only suggestive of a P_2X7_R-A_2A_R interaction, this contention is further supported by the independent *in vitro* experiments showing that P_2X7_R and A_2A_R closely interact in the control of calcium responses in N9 microglial cells. This indicates that these two, so far considered independent, arms of the purinergic system (Agostinho et al., 2020), operated by ATP-P_2_R and by adenosine-P_1_R might actually cooperate to control adaptative brain function. Importantly, this proof-of-concept, so far only confirmed to occur in male rats (selected to cope with the “3R” guidelines), still needs to be extended to female rats, an issue of particular importance since there are gender differences in the A_2A_R modulation of microglia and neuroinflammatory-like responses in rodents (Caetano et al., 2017; Simões-Henriques et al., 2020).

The present study extends to a model of repeated restraint stress the ability of P_2X7_R blockade to attenuate behavioral modifications upon chronic stress (Iwata et al., 2016; Yue et al., 2017; Farooq et al., 2018; Aricioglu et al., 2019; reviewed in Illes et al., 2020). This is in agreement with the association of P_2X7_R polymorphisms with depressive symptoms (see meta-analysis in Czamara et al., 2018) and reinforces the concept of ATP as a danger signal in brain dysfunction (reviewed in Rodrigues et al., 2015). As observed by others in different animal models of brain dysfunction (Jimenez-Pacheco et al., 2013; Wang et al., 2017; Martínez-Frailes et al., 2019; Song et al., 2019), namely upon chronic stress (Yue et al., 2017; Dang et al., 2018; but see Kongsui et al., 2014), we identified an up-regulation of P_2X7_R and an ability of P_2X7_R to control different markers of neuroinflammation, as also reported in other animal models of depression (Yue et al., 2017; Bhattacharya and Jones, 2018), to mediate stress-induced behavioral modifications (Rial et al., 2016; Deng et al., 2020; Troubat et al., 2021).

The present study also provides the first demonstration that a prolonged (days) intake of caffeine prevents behavioral modifications caused by repeated restraint stress, as has been observed in other animal models of stress (Pechlivanova et al., 2012; Kaster et al., 2015; Yin et al., 2015; Kasimay Cakir et al., 2017) and in individuals with mood dysfunction, namely depression (reviewed in Grosso et al., 2016; Wang et al., 2016) and suicide ideation (e.g., Lucas et al., 2014; Park et al., 2019). The protective effects of caffeine in animal stress models are mimicked by selective A_2A_R blockade (Kaster et al., 2015) and A_2A_R polymorphisms are associated with the incidence of major depression (Oliveira et al., 2019). We also observed an up-regulation of A_2A_R, as occurs in different conditions of brain dysfunction (reviewed in Cunha, 2016), namely upon repeated stress (Cunha et al., 2006; Kaster et al., 2015). A_2A_R, as well as caffeine, can control abnormal synaptic plasticity and synaptic dysfunction (e.g., Kaster et al., 2015; Temido-Ferreira et al., 2020) and also control microglia reactivity and neuro-inflammation (e.g., Brothers et al., 2010; Rebola et al., 2011; Mao et al., 2020), but the exact mechanism underlying the ability of A_2A_R to control mood dysfunction upon chronic stress remains to be defined.

Apart from establishing the ability of BBG and caffeine to attenuate behavioral alterations in this particular model of repeated restraint stress, the major finding of the present study is the existence of putative crosstalk between the two purinergic signaling systems operated by each of these antagonists. The inhibition of the stress-induced up-regulation of A_2A_R by BBG and, conversely, the inhibition of the stress-induced up-regulation of P_2X7_R by caffeine is suggestive of crosstalk between the two types of purinergic receptors *in vivo*. This was reinforced by parallel experiments studying calcium transients in microglial N9 cells. In fact, in microglial N9 cells, A_2A_R activation increased and A_2A_R blockade decreased BzATP-induced calcium transients, which was mediated by P_2X7_R, and conversely, a selective P_2X7_R antagonist attenuated CGS26180-induced calcium transients, which was largely mediated by A_2A_R. Since synaptic alterations have also been proposed to underlie stress-induced alterations of brain function (Duman and Aghajanian, 2012; Vose and Stanton, 2017), we also investigated if there was crosstalk between P_2X7_R and A_2A_R in synaptic alterations, namely in the process of LTP in the hippocampus. While we have previously established a selective role of A_2A_R controlling synaptic plasticity without an effect on basal synaptic transmission (Costenla et al., 2011; Gonçalves et al., 2019; Temido-Ferreira et al., 2020), a putative role of P_2X7_R on the control of hippocampal synaptic transmission has been controversial (Armstrong et al., 2002; Kukley et al., 2004; Klaft et al., 2012; Khan et al., 2019) and an eventual role of P_2X7_R on the control of synaptic plasticity had not yet been tested. We now show that BzATP decreases synaptic transmission, but this effect is blocked by the selective A_1_R antagonist DPCPX (see Kukley et al., 2004), following the remarkable efficiency of ectonucleotidases to metabolize ATP derivates into their adenosine derivative counterparts (Cunha et al., 1998) to activate the abundant and efficient presynaptic A_1_R that decrease excitatory transmission in the hippocampus (reviewed in Dunwiddie and Masino, 2001). Thus, we resorted to testing the impact of P_2X7_R antagonists (BBG and JNJ47965567) on hippocampal LTP and concluded that P_2X7_R does not seem to control hippocampal LTP under physiological conditions. Furthermore, we did not observe the ability of P_2X7_R antagonists to modify the decrease of LTP caused by the blockade of A_2A_R.

In contrast to the inconclusive effects on a putative P_2X7_R-A_2A_R interaction in the control of synaptic plasticity, the crosstalk between P_2X7_R and A_2A_R in the control of microglial responses suggests that the interplay between P_2X7_R and A_2A_R to control brain maladaptive function upon repeated stress might mostly be due to crosstalk in the control of neuroinflammation rather than of synaptic plasticity. Interestingly, crosstalk between P_2_ and P_1_ receptors in the control of microglia was first documented by Kettenmann’s group (Färber et al., 2008) and further developed by Koizumi’s group (reviewed in Koizumi et al., 2013); however, these P_2_R-P_1_R interactions in microglia were not characterized to involve P_2X7_R and A_2A_R, although parallel effects of P_2X7_R and A_2A_R have previously been described to control inflammatory processes (Savio et al., 2017) and brain injury (Ye et al., 2018). We now demonstrate direct crosstalk between both receptors in the control of microglial N9 cell responses, which is paralleled by the ability of antagonists of each receptor to control the other’s up-regulation upon repeated stress. This is highly suggestive of direct cooperation between the two arms of the purinergic modulation system to control neuro-inflammation and the adaptive central responses to repeated stress. However, future studies still need to detail if the P_2X7_R-A_2A_R interaction only occurs in microglia or might also take place in astrocytes. In fact, P_2X7_R (reviewed in Franke et al., 2012) and A_2A_R (reviewed in Cunha, 2016) also have profound effects on the pathophysiological roles of astrocytes and the involvement of astrocytes in the control neuroinflammation and neuronal function as well as adaptation to repeated stress (reviewed in Rial et al., 2016) cannot exclude them as a possible major locus of P_2X7_R-A_2A_R interactions to control the observed behavioral modifications upon repeated restraint stress.

The detailed mechanisms of this P_2X7_R-A_2A_R interactions also remain to be unraveled and they can involve different possibilities: one possibility is the formation of heteromers, which has been documented for P_2X7_R (Antonio et al., 2011) and for A_2A_R (reviewed in Ferré and Ciruela, 2019) and between different P_2_R and P_1_R (Namba et al., 2010); another possibility is the use of transducing systems of each receptor to control the other receptor function, as has been shown for P_2X7_R controlling metabotropic receptors (reviewed in Miras-Portugal et al., 2019), A_2A_R controlling ionotropic receptors (e.g., Garção et al., 2013; Temido-Ferreira et al., 2020) and between different P_2_R and P_1_R (George et al., 2016); a third possibility is a key role of ecto-nucleotidases metabolizing ATP into adenosine in a rapid (Dunwiddie et al., 1997; Cunha et al., 1998) and highly controlled manner (James and Richardson, 1993; Cunha, 2001) to format the balanced activation of both receptors (Kukley et al., 2004; Liston et al., 2020). After this first step establishing an interaction between A_2A_R and P_2X7_R, future work will be required to detail the mechanistic basis of this A_2A_R-P_2X7_R interaction.

In conclusion, the present study provides evidence for crosstalk between P_2X7_R and A_2A_R in the control of neuroinflammation and adaptive responses to restraint stress. The importance of these findings is best heralded by the new prospects to simultaneously target P_2X7_R and A_2A_R to maximize the neuroprotective potential of the purinergic system. The present findings place at the center-stage the need to study the purinergic system as a whole and understand the relative contribution of its different constituents to provide the required integrative views (see Agostinho et al., 2020) to justify robust protective strategies to control maladaptation of brain function characteristic of neuropsychiatric disorders.

## Data Availability Statement

Data will be made available upon reasonable and justified request. Requests to access the datasets should be directed to cunharod@gmail.com.

## Ethics Statement

The animal study was reviewed and approved by the Portuguese Ethical Committee (DGAV) and by the Institution’s Ethics Committee (ORBEA 238-2019/14102019).

## Author Contributions

LD and AT carried out the Ca transient experiments in N9 cells. CL and FG carried out the electrophysiological recordings. AN, DP, and NM carried out the behavioral experiments. PA and RC coordinated the project and wrote the manuscript. All authors contributed to the article and approved the submitted version.

## Conflict of Interest

RC is a scientific consultant of the Institute for Scientific Information on Coffee (ISIC). The remaining authors declare that the research was conducted in the absence of any commercial or financial relationships that could be construed as a potential conflict of interest.
